# Consensus versus Individual QSARs in Classification: Comparison on a Large-Scale Case Study

**DOI:** 10.1021/acs.jcim.9b01057

**Published:** 2020-02-19

**Authors:** Cecile Valsecchi, Francesca Grisoni, Viviana Consonni, Davide Ballabio

**Affiliations:** † Milano Chemometrics and QSAR Research Group, University of Milano Bicocca, P.za della Scienza 1, 20126 Milano, Italy; ‡ Department of Chemistry and Applied Biosciences, ETH Zurich, Vladimir-Prelog-Weg 4, 8049 Zurich, Switzerland

## Abstract

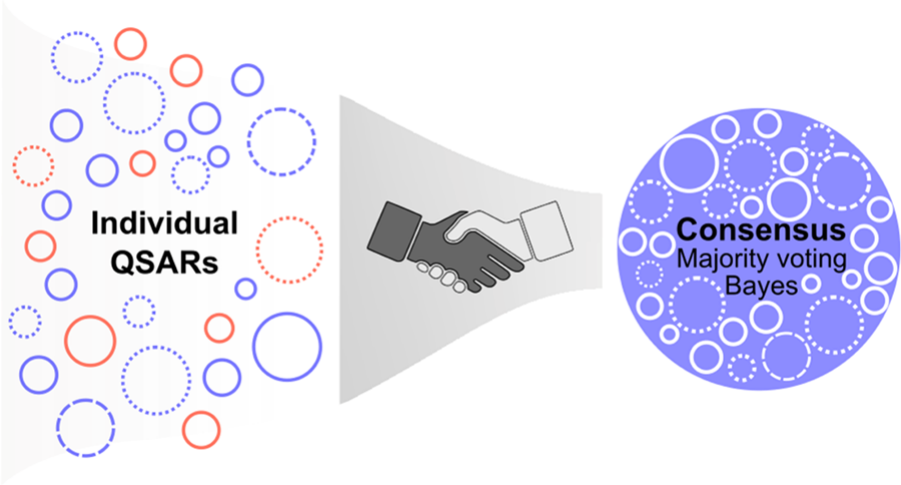

Consensus strategies have been widely applied in many different scientific fields, based on the assumption that the fusion of several sources of information increases the outcome reliability. Despite the widespread application of consensus approaches, their advantages in quantitative structure–activity relationship (QSAR) modeling have not been thoroughly evaluated, mainly due to the lack of appropriate large-scale data sets. In this study, we evaluated the advantages and drawbacks of consensus approaches compared to single classification QSAR models. To this end, we used a data set of three properties (androgen receptor binding, agonism, and antagonism) for approximately 4000 molecules with predictions performed by more than 20 QSAR models, made available in a large-scale collaborative project. The individual QSAR models were compared with two consensus approaches, majority voting and the Bayes consensus with discrete probability distributions, in both protective and nonprotective forms. Consensus strategies proved to be more accurate and to better cover the analyzed chemical space than individual QSARs on average, thus motivating their widespread application for property prediction. Scripts and data to reproduce the results of this study are available for download.

## 1 Introduction

Consensus approaches aim to combine and integrate information derived from different sources to increase the outcome reliability and overcome limitations of single approaches.^1^ In the framework of quantitative structure–activity relationships (QSARs), they are generally recognized as valuable tools to reduce the effects of underestimating uncertainties in the prediction of biological activities.^2,3^

The main underlying assumption of consensus modeling in QSAR is that individual models, due to their reductionist nature, consider only partial structure–activity information, as encoded by molecular descriptors and adopted algorithms. Thus, the combination of multiple QSAR predictions may provide a wider knowledge and increase the reliability associated with the predictions compared to individual models.^1,4^ Indeed, one of the advantages of the consensus methods is the reduction of the effects of contradictory information by averaging the predictions of models,^1,5−8^ although this is not always reflected in improvements of the predictive ability compared to single models.^1,5^ Furthermore, integrating individual QSARs can broaden the applicability domain, that is, the chemical space where predictions can be considered reliable.^9,10^ For these reasons, consensus methods, also known as high-level data fusion or ensemble approaches, have been extensively applied in QSAR studies.^11−16^ Recent studies on the improvement achieved with large-scale consensus approaches for quantitative (regression) models can be found in the literature.^17,18^ However, to the best of our knowledge, no thorough evaluation of the consensus versus single qualitative (classification) model performance has been carried out to date, since only a few QSAR models are usually available for the same endpoint.^6,10,19−23^

The present study was based on the outcome of a large collaborative project (Collaborative Modeling Project of Androgen Receptor Activity, CoMPARA^19^), which produced three data sets containing experimental values on androgen receptor (AR) modulation and corresponding QSAR predictions, namely, (i) binding to AR (34 QSAR models), (ii) AR antagonism (22 QSAR models), and (iii) AR agonism (21 QSAR models).^19^ CoMPARA was chosen as a test system due to the large availability of diverse QSAR-based predictions. Note that in the framework of CoMPARA, two ad hoc consensus approaches were applied by combining predictions with a weighting score based on the goodness-of-fit, predictivity, and robustness of models.^24^ However, the aim of the present study is not a comparison with these former consensus approaches, which were specifically targeted to screen and prioritize chemicals for endocrine activity, but the systematic investigation of the advantages of further consensus strategies compared to single QSAR models. To this end, approaches with varying levels of complexity (majority voting and Bayesian methods, in both protective and nonprotective versions) were considered. Moreover, we investigated whether the exclusion of the worst-performing models may influence the consensus outcome, in terms of chemical space coverage and predictive performance.^13,15^ Finally, a structural similarity analysis was carried out to identify specific chemical regions where individual QSAR models, and the respective consensus outcome, fail in their predictions.

## 2 Materials and Methods

### 2.1 Collaborative Project

The QSAR models considered in this work were previously developed in the framework of a collaborative project (Collaborative Modeling Project of Androgen Receptor Activity, CoMPARA^24^), coordinated by the National Center of Computational Toxicology (U.S. Environmental Protection Agency). CoMPARA aimed to develop in silico approaches to identify potential androgen receptor (AR) modulators. This project involved 25 research groups worldwide, which were provided with a calibration set consisting of 1689 chemicals and the corresponding experimental annotations on binding, agonism, and antagonism activities (in the form of qualitative labels, active/inactive), as determined by a battery of 11 in vitro assays.^20^ The research groups were then asked to predict another 55 450 chemicals for one or more endpoints (binding, agonism, and antagonism) using their own developed QSAR models. Finally, these predictions were merged through ad hoc consensus approaches, which are currently being used by the CoMPARA coordinators to prioritize experimental tests for potential endocrine-disrupting chemicals.^24^

The predictive ability of individual QSAR models was assessed by the project coordinators on the basis of three specific evaluation sets, which were embedded within the large prediction set of 55 450 chemicals, to carry out a blinded verification. These sets were created from literature data extracted from different sources and curated for quality, by considering target, modality, hit call, and concordance among the annotated values. The three evaluation sets included 3540 chemicals annotated with binding activities, 4408 with agonism, and 3667 with antagonism. We used the individual QSAR predictions for these three evaluation sets, whose details are summarized in Table 1, to calculate the consensus approaches. All evaluation sets are characterized by unbalanced sample distribution toward inactivity with 88.4, 91.4, and 96.3% of inactive chemicals for binding, antagonism, and agonism, respectively. The three evaluation sets, including chemical identifiers, SMILES, and predictions, are available as the Supporting Information describing the CoMPARA project.^24^

**Table 1:** Number of Chemicals (Total, Actives, and Inactives) Included in the CoMPARA Binding, Antagonism, and Agonism Evaluation Sets and Number of Models Developed within the CoMPARA Project for Each Endpoint

	binding	antagonism	agonism
number of chemicals	3540	3667	4408
active	411 (11.6%)	314 (8.6%)	164 (3.7%)
inactive	3129 (88.4%)	3353 (91.4%)	4244 (96.3%)
individual QSAR models	34	22	21

Note that although the project coordinators also provided quantitative binding, agonism, and antagonism activities, the participants developed only a few regression models (five, five, and three for binding, agonist, and antagonist, respectively). We considered, thus, only classification models for consensus approaches to allow for a comprehensive and systematic analysis.

### 2.2 Individual QSAR Models

CoMPARA consortium members trained QSAR models to classify chemicals for their potential of AR binding (34 models), agonism (21 models), and antagonism (22 models). Models were mainly developed on the same calibration set of 1689 chemicals, using different modeling strategies (e.g., artificial neural networks, *k*-nearest neighbors, support-vector machines, partial least squares discriminant analysis, classification trees^8,22,23^) and molecular descriptors (e.g., binary fingerprints and nonbinary descriptors).^24^ Each submitted prediction was associated with the applicability domain (AD) assessment, that is, an indication on whether predictions can be considered as reliable.^9,25^

**Figure 1:**
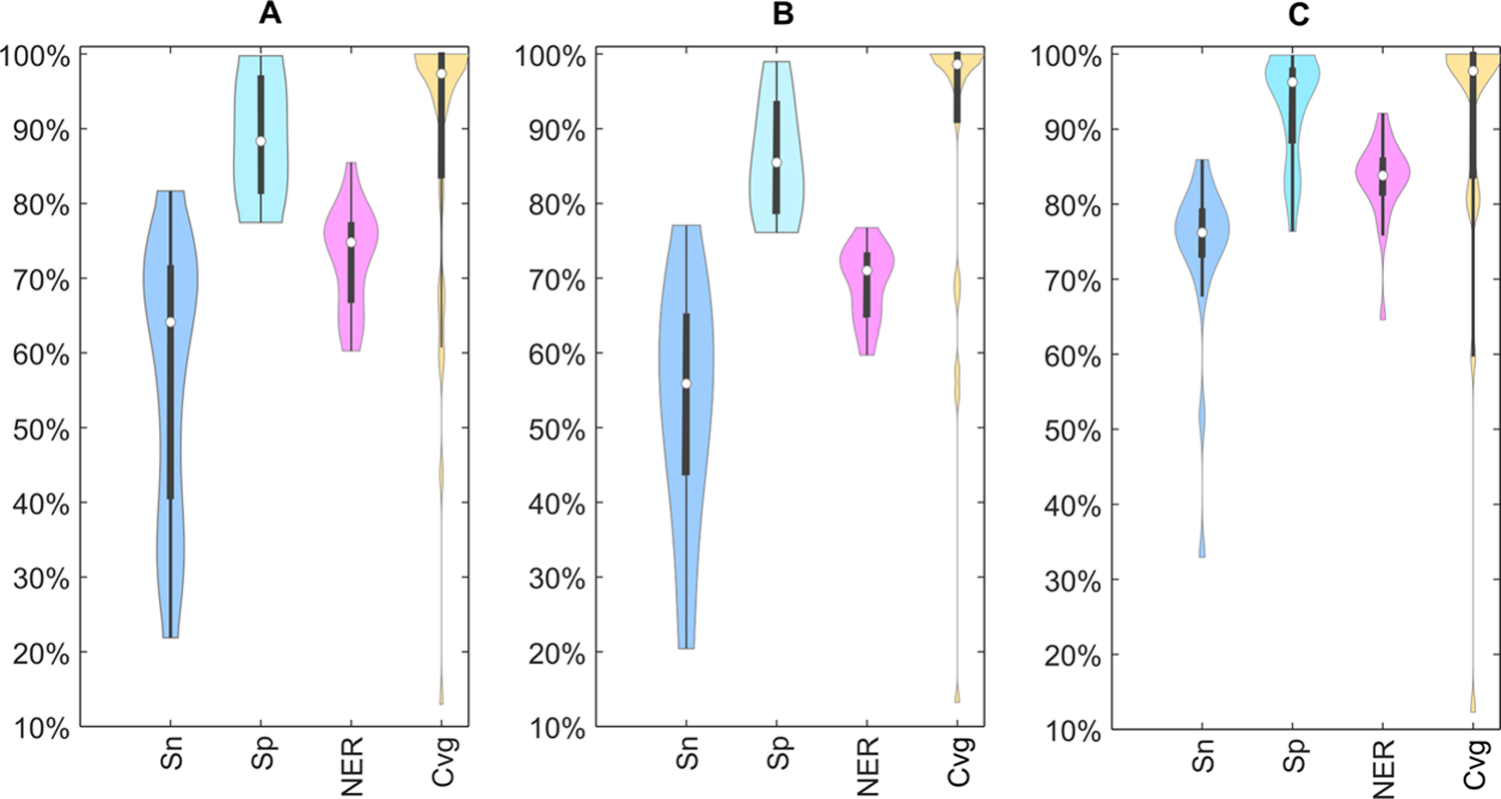
Violin plots of sensitivity (Sn), specificity (Sp), non-error rate (NER), and coverage (Cvg) for the individual CoMPARA models on the binding (A), antagonism (B), and agonism (C) evaluation sets. Empty dots indicate median values, thick gray lines indicate the second and third quartiles, and thin gray lines indicate the first and fourth quartiles. Shapes indicate the underlying data distribution. Numerical values of the classification parameters for all of the models are provided in the Supporting Information (Tables S1–S3).

The predictive ability of QSAR models was assessed on the evaluation set through the following classification measures: (i) sensitivity (Sn) and specificity (Sp), which are the percentages of correctly classified active and inactive chemicals, respectively, and (ii) the non-error rate (NER), also known as balanced accuracy, that is the average of Sn and Sp.^26^ Moreover, the percentage of reliably predicted chemicals (coverage, Cvg) was used as an additional criterion to assess the model performances. The distribution of the classification estimators of the individual CoMPARA models for the three modeled endpoints is summarized in Figure 1.

All models have a good predictive performance, with the median NER ranging from 71.0% (antagonism) to 83.8% (agonism). Specificity values (Sp) are always higher than sensitivities (Sn), thus indicating a better performance of the models in the identification of inactive compounds. Except for the agonism endpoint, sensitivity is associated with a higher variability than specificity, with values ranging from ∼20 to ∼80% on both binding (relative standard deviation equal to ∼28%) and antagonism (relative standard deviation equal to ∼29%) endpoints. This general behavior can be due to both unbalanced classes, which are strongly skewed toward inactivity (88.4 and 91.4% of inactive molecules for binding and antagonism data sets, respectively; Table 1), and differences in the ranges of testing between training and evaluation sources, as reported in the literature.^24^

The models for agonism show the best trade-off between sensitivity (Sn) and specificity (Sp), with most models characterized by sensitivity values in the range of ∼70 to ∼84% and specificity in the range of ∼76 to ∼100%. Additionally, agonism models have the highest median sensitivity (76.2%), specificity (96.3%), and NER (83.8%), although the agonism data set includes only 3.7% of actives and is thus the most unbalanced among the three evaluation sets (see Table 1). Models for binding and antagonism have similar median NERs (74.8 and 71%, respectively), moderately low median sensitivities (64.1 and 55.9%), and high median specificities (88.3 and 85.5%).

The majority of individual models are characterized by a high percentage of reliably predicted chemicals (coverage values equal to 88.1, 88.1, and 89.5% on average for binding, antagonism, and agonism, respectively). The models that are able to reliably predict only a few molecules are associated with the highest classification performance, thus confirming that high classification performance is more likely on a narrow applicability domain. In fact, the four best models to predict the binding activity (NER higher than 80%) were characterized by a limited percentage of chemicals in their applicability domain (coverage values equal to 13, 43.7, 60.7, and 69%; Table S1), suggesting that these single models have limited applications for prioritization purposes.

### 2.3 Consensus Methods

In this study, two consensus strategies were applied to integrate the predictions provided by individual models: majority voting and the Bayes consensus with discrete probability distributions. These methods are briefly described below.

#### 2.3.1 Majority Voting

Voting methods combine the predictions provided by independent models with different frequency-based strategies, such as averaging and scoring.^14,16,23,27^ The most simple and intuitive voting approach is the majority voting (MV) rule, which assigns a chemical to the most frequently predicted class among the pool of considered models.^28,29^ Cautionary (protective) voting approaches can be obtained by considering only predictions integrated with a sufficiently high concordance (based on a user-defined threshold) among the pool of models.

In this work, we considered three different majority voting strategies as follows: (i) majority voting loose (MVL), (ii) majority voting intermediate (MVI), and (iii) majority voting strict (MVS). The “loose” approach classifies molecules using the most recurrent class assignment. In the two-class case, this corresponds to the class predicted with a frequency higher than 50%. The “intermediate” and “strict” criteria (MVI and MVS, respectively) are protective approaches. MVS assigns the compound only if the prediction agreement is higher than or equal to 75%. The MVS approach provides a prediction for a given molecule only if all of the individual models predict the same class (100% agreement). To ensure the reliability of the consensus outcome, only the predictions within the applicability domain of individual models were considered for the calculation of the agreement.

#### 2.3.2 Bayesian Consensus

An alternative to the majority voting approach is a probabilistic method, such as Bayesian consensus. The Bayes rule,^12,30,31^ in particular, estimates the prior probability for a molecule to belong to a specific class for each information source and then combines this information to provide a joint probability.^32^

In particular, the Bayes consensus with discrete probability distributions^31,33^ initially takes into account the first evidence, *e*, which is in this case the class (active or inactive) predicted by the first model. Then, the posterior probabilities *p*(*h_g_*|*e*) that hypothesis *h_g_* is true given evidence *e* are calculated for any class *g*, as follows
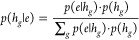
1where *p*(*e*|*h*_*g*_) is the likelihood probability that evidence *e* is observed given that hypothesis *h*_*g*_ is true and *p*(*h*_*g*_) is the prior probability that hypothesis *h*_*g*_ is true in the absence of any specific evidence.

With two hypotheses (i.e., class equal to “active” or “inactive”), the prior equal (noninformative) probability is estimated as *p*(*h*_ACTIVE_) = *p*(*h*_INACTIVE_) = 0.50. The prior proportional (informative) probability for each hypothesis *h*_*g*_ would be *p*(*h*_*g*_) *= n*_*g*_/*n*, where *n*_*g*_ is the number of molecules belonging to the *g*th experimental class within the *n* total molecules.

Likelihood probabilities for each model can be estimated from its confusion matrix, where the numbers of correct and incorrect classifications are collected.^31^ Once posterior probabilities for the first model have been calculated, the Bayes consensus proceeds with the following iterative procedure. Posterior probabilities of the first model are used as new prior probabilities for the second step, where the class predicted by the second model is the new evidence e on the basis of which the posterior probabilities are calculated. These posterior probabilities become the new prior probabilities in the third iteration and so on, until predictions of all models have been used in the consensus process. At the end of the iterations, the posterior probabilities corresponding to the combination of all of the information sources are obtained. Therefore, the Bayes consensus assigns a probability value to each class, which is then used for prediction, by choosing the class with the maximum posterior probability. As for the majority voting strategies, the Bayes consensus can be used in a protective manner by setting a posterior probability threshold (in this study, 95%) that has to be fulfilled to predict the class.^31^

When proportional prior probabilities are used with models calibrated on data with unbalanced class distributions, Bayes results may change depending on which model sequence enters the iteration process. In fact, if models are associated with different prior proportional probabilities, the model entering the first position of the iterative process can produce a different outcome with respect to others. This is the case of the collaborative project under analysis, whose models were calibrated and validated on the same set of chemicals, but with different ratios of molecules included in the applicability domain, leading to different prior probabilities. To overcome this potential issue, in this study, we used equal prior probabilities.^11^

As for the majority voting approaches, predictions associated with molecules outside the applicability domain of individual models were not considered.

### 2.4 Analysis of Molecular Similarities

A molecular similarity analysis was carried out to investigate potential relationships between the molecular structure and misclassifications provided by QSAR and consensus models. To this end, extended connectivity fingerprints (ECFPs),^34^ which encode for the presence of branched substructures in a binary array, were used as molecular descriptors, with the setting specified in Section 2.5. Pairwise molecular similarities, as quantified using the Jaccard–Tanimoto similarity coefficient,^35^ were used to produce a two-dimensional representation of the molecular space by means of multidimensional scaling (MDS).^36^

### 2.5 Software

ECFP04 (1024 bits and 0–2 bond radius) were calculated by means of DRAGON 7^37^ with default settings (“Bits per pattern” = 2; “Count fragments”: True; “Atom Options”: [Atom type, Aromaticity, Connectivity total, Charge, Bond order]). MDS was carried out in MATLAB 2018b^38^ by a publicly available toolbox.^39^ Consensus strategies were performed using the MATLAB code written by the authors, which is available for download at http://www.michem.unimib.it/download/data/bayes-and-majority-voting-consensus-for-matlab/. Violin plots were created with the code available at the URL https://github.com/bastibe/Violinplot-Matlab.

## 3 Results

### 3.1 Analysis of Consensus Strategies

#### 3.1.1 Classification Performance

The selected consensus strategies (i.e., Bayes [B], protective Bayes [Bp], majority voting loose [MVL], majority voting intermediate [MVI], and majority voting strict [MVS]) were used to integrate the predictions of the individual QSAR models for binding, antagonism, and agonism. When applying protective consensus strategies, the outcome predictions were rejected if related to potential uncertainty, that is, (i) prediction agreement lower than 75 and 100% for MVI and MVL, respectively, and (ii) posterior probability lower than 95% for protective Bayes. For majority voting loose (MVL), no prediction was provided in the case of equal frequency for the two classes (50%).

**Figure 2:**
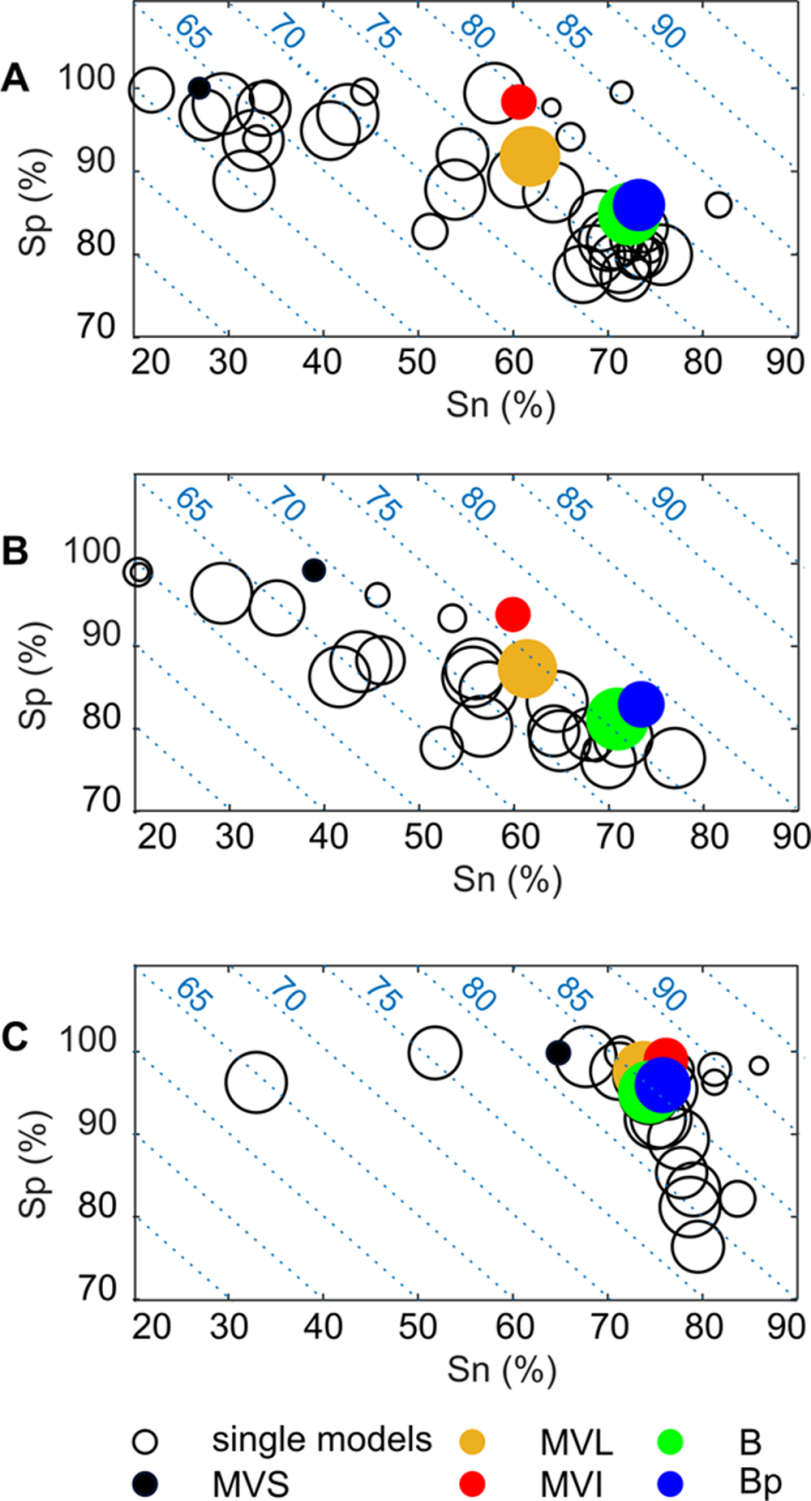
Plot of sensitivities (Sn) versus specificities (Sp) for the individual models (black empty circles) and for the consensus approaches (filled, colored circles) for each endpoint: (A) binding, (B) antagonism, and (C) agonism. Green, blue, red, yellow, and black circles indicate Bayes (B), Bayes protective (Bp), majority voting intermediate (MVI), loose (MVL), and strict (MVS) consensus, respectively. The size of the circles is proportional to the coverage (Cvg); the smaller a circle, the lower the coverage. Isolines represent NER variations (5% steps).

In analogy with the individual models, the consensus approaches were evaluated for their classification performance, in terms of sensitivity (Sn), specificity (Sp), non-error rate (NER), and coverage (Cvg) (Table 2). A graphical comparison with individual models is represented in Figure 2 with plots of sensitivity versus specificity values. Moreover, since sensitivity, specificity, and coverage have the same unit scale and optimality direction (i.e., ranging from 0 to 100%; the closer to 100%, the better), a comprehensive performance index was calculated as their arithmetic average, denoted as “Utility” in the framework of ranking analysis and multicriteria decision making.^40−43^ Both consensus and individual QSARs were ranked for decreasing values of Utility (Table 2).

**Table 2:** Classification Performance of the Consensus Approaches for Binding, Agonism, and Antagonism Endpoints^a^^a^

	binding (34 models)					antagonism (22 models)					agonism (21 models)				
consensus approach	Sn (%)	Sp (%)	NER (%)	Cvg (%)	rank	Sn (%)	Sp (%)	NER (%)	Cvg (%)	rank	Sn (%)	Sp (%)	NER (%)	Cvg (%)	rank
MVL	61.8	91.8	76.8	99.3	4	61.5	87.3	74.4	98.9	3	73.8	97.5	85.7	99.7	2
MVI	60.6	98.3	79.5	80.6	8	60.0	93.8	76.9	80.1	4	76.1	99.0	87.5	91.5	6
MVS	26.9	100	63.5	37.5	39	39.0	99.2	69.1	42.4	25	64.8	99.9	82.3	51.4	17
B	72.3	84.9	78.6	100	1	71.0	81.2	76.1	100	1	74.4	95.1	84.7	100	3
Bp	73.3	85.9	79.6	96.1	7	73.5	82.9	78.2	92.9	2	75.8	95.9	85.9	97.7	4

a For each consensus approach, sensitivity (Sn), specificity (Sp), non-error rate (NER), coverage (Cvg), and total ranking are reported. B, Bayes; Bp, protective Bayes; MVL, majority voting loose; MVI, majority voting intermediate; MVS, majority voting strict.

Consensus strategies have better NERs than individual QSARs on average, without substantial losses in terms of coverage compared to individual models; additionally, consensus models are always ranked among the top 10 positions (Table 2). The exception is MVS, which provides a remarkably lower coverage (lower than 52% for all of the endpoints), due to the required 100% agreement among multiple predictions (up to 34 predictions). The narrow coverage of MVS, however, was not counterbalanced by a better performance compared to the other consensus approaches. MVS, in fact, showed the lowest NER and Cvg values among all of the tested consensus strategies. For these reasons, MVS was not analyzed further in this framework.

Unlike MVS, the other consensus strategies generally showed a better trade-off between the classification performance and the chemical space coverage than individual QSARs. For instance, the two single-binding models in the upper-right region of the sensitivity versus specificity space (Figure 2A) have the best predictive performance for binding, with NERs equal to 85.5 and 83.8%, respectively (Table S1), but they cover only a small portion of the chemical space, as it results from the small coverage values (43.7 and 60.7%, respectively). On the other hand, the protective Bayes (Bp) reached a slightly lower NER (79.6%) but higher coverage (96.1%).

The models on binding and antagonism (Figure 2A,B) endpoints are characterized by the unbalanced specificity and sensitivity values, with several models showing high specificity (Sp > 90%) and low sensitivity (Sn < 50%). For these endpoints, consensus methods achieved more balanced values of sensitivity and specificity, due to the compensation in the integration of diverse sources of information. This is particularly evident in the case of the Bayes approaches (Table 2), ranked as the best overall approach for both binding and antagonism, and confirms that the uncertainty can be reduced by the integration of conflicting sources.

The difference in the performance between consensus and individual QSARs is less pronounced when considering agonism (Figure 2C), since the individual models have more homogeneous NERs and balanced Sn and Sp values compared to the other case studies. Therefore, consensus methods converged to similar performances.

Majority voting approaches inherit the high specificity values of individual models for both binding and antagonism endpoints, while the Bayes consensus led to a higher sensitivity. This trend could be caused by the low false-positive rates of individual models (Figure 1) and the way this information is weighted and integrated into the Bayes calculation (eq 1). Thus, in this framework, if a compound is predicted with an equal frequency as active and inactive by the individual models, it will be more likely assigned to the active class by the Bayes consensus.

Protective approaches (MVI and Bp) yielded slightly better results in terms of the classification performance (NER) compared to their nonprotective counterparts, but with a relatively larger loss in coverage (up to 18.7% loss), especially when dealing with majority voting schemes. This explains the worse position within the ranking of protective approaches with respect to nonprotective ones (Table 2). As an example, the MVL approach on the binding endpoint led to an NER of 76.8% and a coverage of 99.3% (rank 4), while the protective MVI led to a slightly higher NER (79.5%) but considerably lower coverage (80.6%) and a worse rank (8).

#### 3.1.2 Chemical Space Analysis

To evaluate potential associations between misclassifications and structural chemical features, compounds were described by extended connectivity fingerprints (ECFPs). A multidimensional scaling (MDS) was then performed to visualize the similarity relationships (as encoded by the Jaccard–Tanimoto similarity coefficients calculated on ECFPs) in a bidimensional plot. This allowed us to analyze the relationship between such a structural representation and the number of models (individual or consensus), providing reliable predictions.

**Figure 3:**
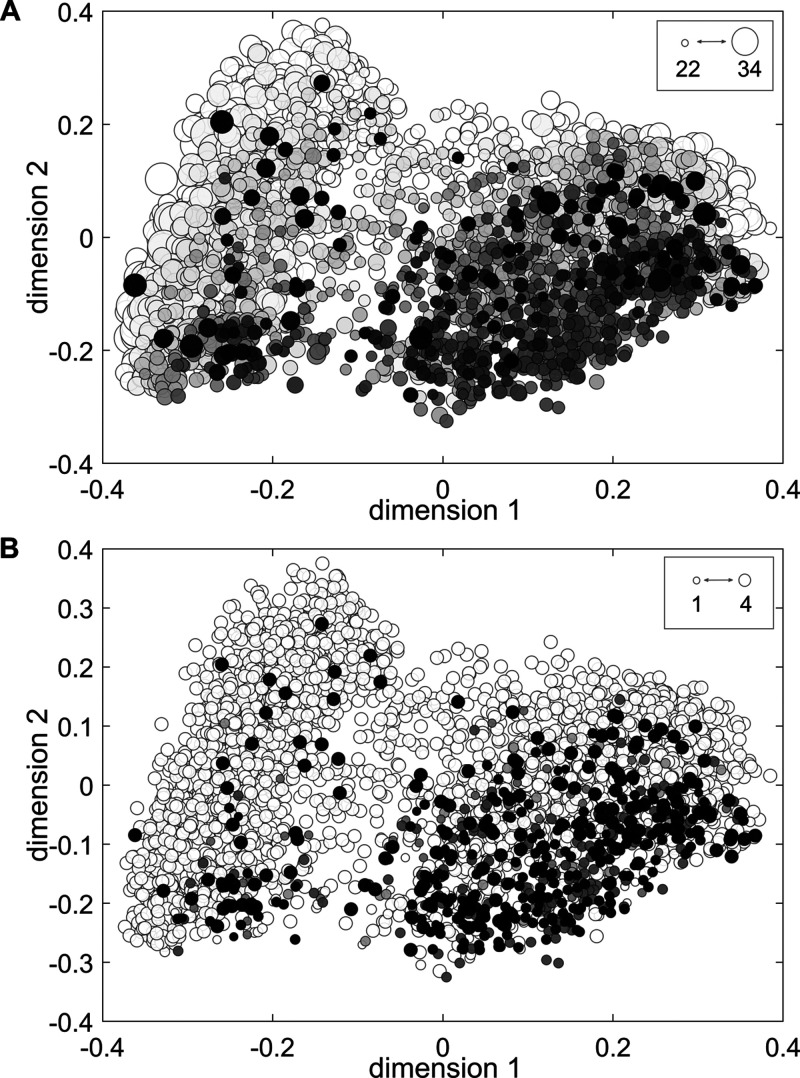
Plot of the first and second dimensions of the MDS for the binding endpoint (ECFPs, Jaccard–Tanimoto similarity). Each point represents a chemical, colored based on the number of misclassifications of (A) individual QSAR models and (B) consensus strategies; the darker the point, the higher the number of misclassifications. The size of each point is proportional to the percentage of models or consensus strategies (A and B, respectively) that provided a prediction for the chemical.

In the obtained MDS representation (Figure 3 for the binding endpoint), chemicals are arranged in two clusters. The cluster characterized by negative scores on the first dimension is mainly composed of aliphatic molecules with long alkyl chains, as well as cyclic aliphatic compounds, mostly with sp^3^-hybridized carbon atoms. The most frequent functional groups are carbonyls, hydroxyls, ethers, and esters, while conjugated structures or p-systems are almost absent in this cluster. The second cluster, located in the positive score region on the first dimension, is mainly composed of conjugated structures, primarily aromatic rings with many electron acceptor substituents (e.g., −NO_2_, −PO_3_, −SO_3_, −F, −Cl, and −CO) and a few donating groups (e.g., −NH_2_ and −OH).

Most of the misclassified molecules cluster in specific regions of the chemical space. Similar distributions were obtained for agonism and antagonism data sets (see Figures S1 and S2). Aliphatic chemicals (characterized by negative scores on the first dimension) are in general well-predicted; on the other hand, misclassifications seem to be mainly grouped in the aromatic cluster (positive scores on the first dimension). Besides incorrect predictions, this region is also associated with lower coverage of the individual models (Figure 3A). Similarly, the intermediate region between the two clusters is characterized by low coverage, reflecting regions of model uncertainty. These observations point toward the presence of relationships between chemical features (as encoded within ECFPs) and model performances, since misclassifications are mainly located in limited portions of the chemical space, where molecules are often out of the models’ applicability domains.

Some chemicals were incorrectly classified by all of the individual QSAR models despite being in their applicability domain, as follows: 19 molecules for binding (all false negatives), 28 for agonism (25 false negatives and 3 false positives), and 37 for antagonism (25 false negatives and 12 false positives). We identified some recurring issues that might explain the observed misclassifications:

1. *Borderline Compounds.* Several active molecules that were consistently predicted as inactive are labeled as having experimental weak or very weak potency (Table S4), as quantified by the half-maximal activity (AC_50_, the molar concentration that produces 50% of the maximum possible activity). The molecules were thus labeled as active, but they actually are borderline between activity and inactivity. Additionally, different activity values due to differences among experimental protocols have been already reported on this set of chemicals.^24^ In such cases, models and experimental data can be regarded as belonging to the same level of assessment^44^ and QSAR models might provide an indication of the potential inactivity of these consistently misclassified compounds.
2. *Differences between Charged and Neutralized Forms*. Another reason could be related to the different activities of charged compounds toward their neutralized counterparts. In fact, traditional QSAR pipelines do not consider annotated counterions and rely on the neutralized form for descriptor calculations. Nine false negatives (two, one, and six for binding, antagonism, and agonism sets, respectively) showed a different activity in their neutralized form and with an annotated counterion (Table S4). For example, 1-butyl-4-methylpyridinium hexafluorophosphate (DTXSID4049296, CASRN 401788-99-6) is a moderate antagonist (AC_50_ = 1.94 μM), but its neutralized form (with removed counterion) is identical to the neutralized forms of 1-butyl-4-methylpyridinium bromide (DTXSID2049345, CASRN 65350-59-6) and 1-butyl-4-methylpyridinium trifluoromethanesulfonate (DTXSID5049368, CASRN 882172-79-4), which are inactive. This highlights the need for considering the effect of charge and counterions on the final biological activity.

Although consensus methods reduced the uncertainty (Figure 3B), misclassifications and unclassified chemicals are still mainly located in the critical region characterized by positive scores of the MDS space (aromatic cluster), thus following the same pattern as individual models. This confirms that consensus approaches can reduce uncertainty but cannot remove it since the integration of erroneous information leads anyway to poor predictions. The performance of consensus models could improve by considering the structural features of chemicals and the individual models’ performance in the chemical space.

### 3.2 Consensus Based on Subsets of Models

**Figure 4:**
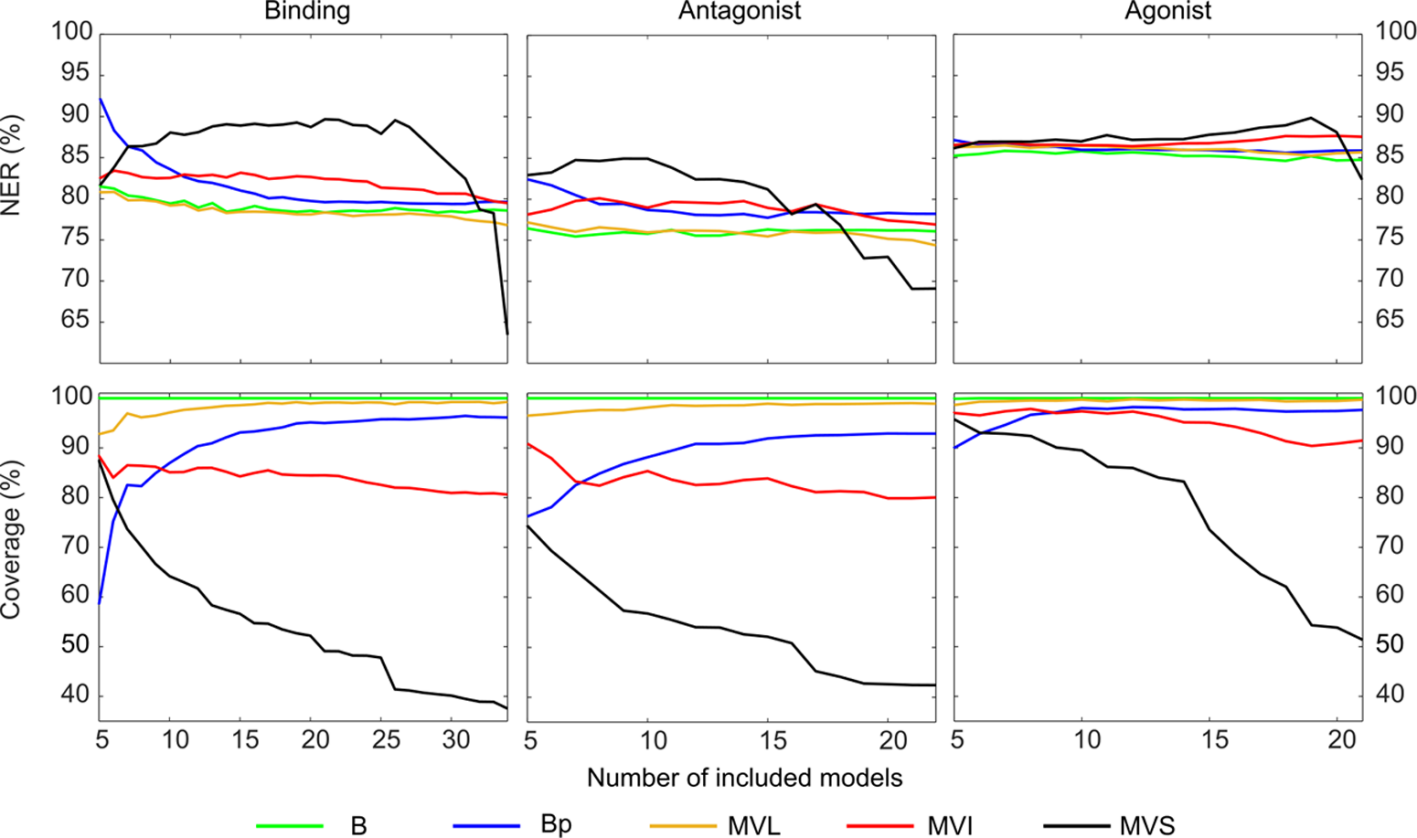
Plot of NER and coverage as a function of the number of models included in the consensus calculation. B, Bayes; Bp, protective Bayes; MVL, majority voting loose; MVI, majority voting intermediate; MVS, majority voting strict.

When integrating several sources of information, one could decide to select only the most reliable ones aiming to neglect misleading information and potentially improve the prediction performance. To this end, we investigated the performance of consensus strategies as a function of the number of merged individual QSARs, ordered by decreasing predictive performance. For each endpoint, subsets of models were selected as inputs for the consensus approaches with the following strategy: (i) the individual QSAR models were ranked according to their NER; (ii) consensus approaches were then calculated iteratively adding one model at a time, starting from an initial subset including the best top five (Figure 4).

The NERs of B, MVI, and MVL are slightly influenced by the number of included models. This indicates that these methods are not sensitive to the integration of poor sources of information in the consensus process. On the contrary, the protective Bayes approach (Bp) is characterized by better performances when a few good models are included, at the expense of the coverage, which shows a considerable decrease. Therefore, when the maximization of the prediction reliability is the only priority, only the most reliable sources of information shall be used in the consensus. When the final goal is to screen a large set of chemicals for testing prioritization, as in the case of the CoMPARA project, the inclusion of all of the available sources of information can considerably enhance the coverage without a significant loss of performance. MVS is the consensus approach showing the highest dependence on the number of included models; in particular, as soon as spurious information sources enter in the consensus process, the coverage significantly decreases.

Table 3 collects the classification performance of consensus approaches calculated on the top five models (chosen based on NER), which is on average better than that of individual models, with consensus strategies occupying the first seven ranking positions for all of the three considered case studies.

**Table 3:** Classification Performance of the Consensus Approaches Estimated on the Binding, Antagonism, and Agonism Sets Considering the Best Five Models Only (Selected Based on NER)^a^

	binding (5 models)					antagonism (5 models)					agonism (5 models)				
consensus approach	Sn (%)	Sp (%)	NER (%)	Cvg (%)	rank	Sn (%)	Sp (%)	NER (%)	Cvg (%)	rank	Sn (%)	Sp (%)	NER (%)	Cvg (%)	rank
MVL	63.9	97.7	80.8	92.8	3	71.6	82.8	77.2	96.5	2	73.8	98.8	86.3	98.6	2
MVI	65.7	99.3	82.5	88.4	4	71.9	84.4	78.1	90.9	3	74.1	99.0	86.5	97.0	4
MVS	63.8	99.5	81.6	87.4	6	78.3	87.6	83.0	74.4	5	73.1	99.2	86.1	95.8	6
B	72.0	91.0	81.5	100	1	73.2	79.7	76.5	100	1	74.4	96.1	85.2	99.9	3
Bp	88.3	96.2	92.2	58.6	7	79.4	85.5	82.4	76.3	4	76.1	98.2	87.1	90.0	7

a For each consensus approach, sensitivity (Sn), specificity (Sp), non-error rate (NER), coverage (Cvg), and total ranking are reported. B, Bayes; Bp, protective Bayes; MVL, majority voting loose; MVI, majority voting intermediate; MVS, majority voting strict.

The protective consensus (Bp, MVI, and MVS) obtained on this reduced pool of models provided higher sensitivities than those based on the integration of all available models (Table 2), especially for binding and antagonism. However, protective approaches are always ranked worse than the nonprotective counterparts. Finally, the performance of MVS improves, since it is easier to reach a 100% prediction agreement with a few input models compared to using the whole set. For example, for binding endpoints, the NER increased from 63.5 to 81.6% and the coverage increased from 37.5 to 87.4%, respectively.

## 4 Conclusions

In this study, we evaluated the extent to which consensus modeling can outperform individual QSARs, by leveraging a large set of QSAR model predictions on androgen receptor binding, agonism, and antagonism. The protective and nonprotective majority voting and Bayes consensus methods were evaluated for their capability to reduce the prediction uncertainty, increase the classification performance, and overcome limitations of individual QSAR models.

The applied consensus strategies provided a better trade-off between the classification performance and the number of reliably predicted chemicals compared to single QSARs. In fact, consensus methods could correctly weigh in and integrate diverse sources of information, leading to balanced values of sensitivity and specificity, as well as to increased coverage compared to the average of individual QSARs. In fact, only a few models could perform better than consensus in terms of classification indices, but they included a limited percentage of chemicals in their applicability domain.

Protective consensus approaches were found to be suitable to incorporate information of less reliable predictions into the final assessment, thereby providing a slightly better classification performance, at the expense of the coverage.

However, consensus strategies were not able to perform well in those critical regions of the chemical space where most of the individual models failed, since the integration of erroneous information leads, by definition, to poor predictions. Implementation of a structure-driven model selection could help overcome these limitations of consensus approaches.

The performance of consensus strategies was finally evaluated as a function of the number of models included in the integration approach. The difference in terms of the classification performance between nonprotective consensus strategies applied to all of the available models and to the subset of the five most reliable ones is on average around 1% of the non-error rate (balanced accuracy). Therefore, the performance of nonprotective strategies was not significantly influenced by the presence of poorly predictive individual models, thus again demonstrating the ability of these methods to weigh in and integrate conflicting information. On the contrary, protective approaches benefit from the selection of the most predictive models.

Our final recommendation is to choose the consensus approaches based on the envisaged model application. For prioritization purposes, where one might want to predict the largest number of compounds possible, we recommend using nonprotective approaches. In this case, since MV and the Bayes consensus lead to comparable performances, MV could be the method of choice, due to the easier implementation and interpretation of the results. When the objective is, instead, to obtain the most accurate estimate possible, at the expense of the covered chemical space, protective methods should be applied on a subset of selected, best-performing models.

## Supporting Information Available

Classification performances of the individual models on the binding, agonist and antagonist evaluation sets. Summary of the molecules which were considered outside the applicability domain or misclassified by all the individual QSAR models. Plot of first and second multidimensional scaling dimensions for the agonism and antagonism sets. (PDF)
