# Supplementary material for: Consensus versus Individual QSARs in Classification: Comparison on a Large-Scale Case Study
Source: J Chem Inf Model. 2020 Feb 19;60(3):1215–23. doi: 10.1021/acs.jcim.9b01057 (PMC7997107; doi:10.1021/acs.jcim.9b01057)
Supplement: Supplementary file 1 — ci9b01057_si_001.pdf [file ci9b01057_si_001.pdf]

Supporting Information:

Consensus versus individual QSARs in classification: comparison on a large-scale case study

*Cecile Valsecchi[1], Francesca Grisoni[2], Viviana Consonni[1], Davide Ballabio[1] **

[1] Milano Chemometrics and QSAR Research Group, University of Milano Bicocca, P.za della Scienza 1-20126, Milano, Italy

[2] ETH Zurich, Department of Chemistry and Applied Biosciences, Vladimir-Prelog-Weg 4, 8049 Zurich, Switzerland

Corresponding Author

*davide.ballabio@unimib.it

Table S1. Classification performances of the individual models (as labelled in [1]) on the binding evaluation set. For each model, sensitivity (Sn) and specificity (Sp) of the active class, non-error-rate (NER) and coverage (Cvg) are reported.

ID	Binding models	Sn (%)	Sp (%)	NER (%)	Cvg (%)
1	ATSDR_IRFMN_1	33.7	97.5	65.6	97.9
2	ATSDR_IRFMN_2	54.7	92.1	73.4	96.9
3	ATSDR_IRFMN_3	70.1	81.6	75.9	99.8
4	DTU	66.1	94.2	80.1	69.0

5	ECUST	27.5	96.8	62.2	96.5
6	EPA_NCCT_1	40.8	94.9	67.8	99.4
7	EPA_NCCT_2	70.7	81.6	76.2	98.4
8	EPA_NCCT_3	34.0	98.9	66.5	80.5
9	EPA_NRMRL_1	67.3	77.6	72.5	99.0
10	EPA_NRMRL_2	53.9	87.8	70.9	99.7
11	FDA_HHS	69.1	84.1	76.6	100
12	IBMC_1	64.2	87.5	75.9	99.9
13	IBMC_2	75.7	80.0	77.8	99.9
14	IDEA	58.0	99.4	78.7	99.9
15	INS_LA	60.6	89.3	74.9	100
16	Jussieu	31.6	88.9	60.3	100
17	LM	74.5	80.5	77.5	58.6
18	MNegri	32.6	93.7	63.2	100
19	NCATS_1	64.0	97.7	80.9	13.0
20	NCATS_2	21.9	99.8	60.8	93.0
21	NCSTATE	42.6	96.8	69.7	100
22	SWETOX_1	72.5	81.8	77.2	91.9
23	SWETOX_2	73.7	80.1	76.9	95.7
24	TARTU_1	71.3	79.0	75.2	100
25	TARTU_2	68.6	79.9	74.3	100
26	TUM	29.4	98.1	63.8	100
27	UFG	51.3	82.8	67.0	83.7
28	UMEA	71.4	99.5	85.5	43.7
29	UNC	71.9	77.5	74.7	96.4
30	UNIBARI	33.0	93.9	63.4	67.8

31	UNIMIB_1	81.7	86.0	83.8	60.7
32	UNIMIB_2	73.2	79.8	76.5	93.3
33	UNISTRA	44.3	99.6	71.9	64.9
34	VCCLAB	73.8	83.2	78.5	95.4
	**Mean**	56.8	88.9	72.8	88.1
	**Min**	21.9	77.5	60.3	13.0
	**Max**	81.7	99.8	85.5	100
	**Median**	64.1	88.3	74.8	97.4

Table S2 Classification performances of the individual models (as labelled in [1]) on the agonism evaluation set. For each model, sensitivity (Sn) and specificity (Sp) of the active class, non-error-rate (NER) and coverage (Cvg) are reported.

ID	Agonism models	Sn (%)	Sp (%)	NER (%)	Cvg (%)
1	DTU	77.3	97.8	87.5	81.4
2	EPA_NRMRL_1	79.5	76.4	77.9	97.0
3	EPA_NRMRL_2	71.2	97.6	84.4	99.0
4	FDA_HHS	76.2	95.5	85.8	100
5	IBMC_1	74.4	94.9	84.6	99.8
6	IBMC_2	78.7	81.2	79.9	99.9
7	INS_LA	73.8	97.1	85.4	100
8	Jussieu	32.9	96.3	64.6	100
9	LM	81.3	96.3	88.8	59.7
10	NCATS_1	85.9	98.3	92.1	12.3
11	NCATS_2	51.8	99.9	75.8	97.3
12	NCSTATE	67.7	99.4	83.5	100
13	SWETOX_1	79.0	83.3	81.2	97.8
14	SWETOX_2	77.8	85.3	81.6	96.6
15	TARTU_1	75.6	92.0	83.8	100
16	TARTU_2	77.4	89.4	83.4	100
17	TUM	75.0	91.9	83.4	100
18	UFG	83.7	82.2	82.9	84.5
19	UMEA	81.3	97.9	89.6	79.7
20	UNISTRA	71.4	99.9	85.6	80.3
21	VCCLAB	74.4	98.0	86.2	95.2
	**Mean**	73.6	92.9	83.2	89.5

	**Min**	32.9	76.4	64.6	12.3
	**Max**	85.9	99.9	92.1	100
	**Median**	76.2	96.3	83.8	97.8

Table S3 Classification performances of the individual models (as labelled in [1]) on the antagonism evaluation set. For each model, sensitivity (Sn) and specificity (Sp) of the active class, non-error-rate (NER) and coverage (Cvg) are reported.

**ID**	**Antagonism models**	**Sn (%)**	**Sp (%)**	**NER (%)**	**Cvg (%)**
1	DTU	53.6	93.4	73.5	67.8
2	EPA_NCCT_1	35.1	94.6	64.9	98.3
3	EPA_NCCT_2	68.1	79.3	73.7	97.4
4	EPA_NRMRL_1	70.1	76.1	73.1	98.1
5	EPA_NRMRL_2	57.4	84.7	71.0	99.0
6	FDA_HHS	64.6	83.3	74.0	100
7	IBMC_1	55.7	86.3	71.0	99.9
8	IBMC_2	77.1	76.4	76.8	99.8
9	INS_LA	56.1	87.3	71.7	100
10	Jussieu	41.7	86.3	64.0	100
11	LM	68.8	77.5	73.1	57.6
12	NCATS	20.7	99.0	59.8	13.2
13	NCSTATE	29.3	96.4	62.9	100
14	SWETOX_1	71.6	78.9	75.3	99.3
15	SWETOX_2	64.3	79.7	72.0	96.6
16	TARTU_1	56.7	80.3	68.5	100
17	TARTU_2	65.0	78.6	71.8	100
18	TUM	43.9	88.1	66.0	100
19	UFG	52.5	77.7	65.1	91.1
20	UMEA	45.7	96.2	71.0	54.5
21	UNISTRA	20.4	99.0	59.7	70.0
22	VCCLAB	46.0	88.3	67.2	95.2
	**Mean**	52.9	85.8	69.4	88.1

	**Min**	20.4	76.1	59.7	13.2
	**Max**	77.1	99.0	76.8	100
	**Median**	55.9	85.5	71.0	98.6

Table S4 Summary of the molecules which were considered outside the applicability domain or misclassified by all the individual QSAR models. FPs and FNs stand for false positive and false negatives, respectively. Number of FN molecules with concentration of half maximal activity (AC_{50}) above 20 µM (borderline compounds) and with at least a correspondent inactive neutralized form are listed.

	FNs				FPs
	Borderline compounds	Differences between charged and neutralized forms	others	Total	Total
**Binding**	12	2	5	19	0
**Antagonism**	14	1	10	25	12
**Agonism**	3	6	16	25	3

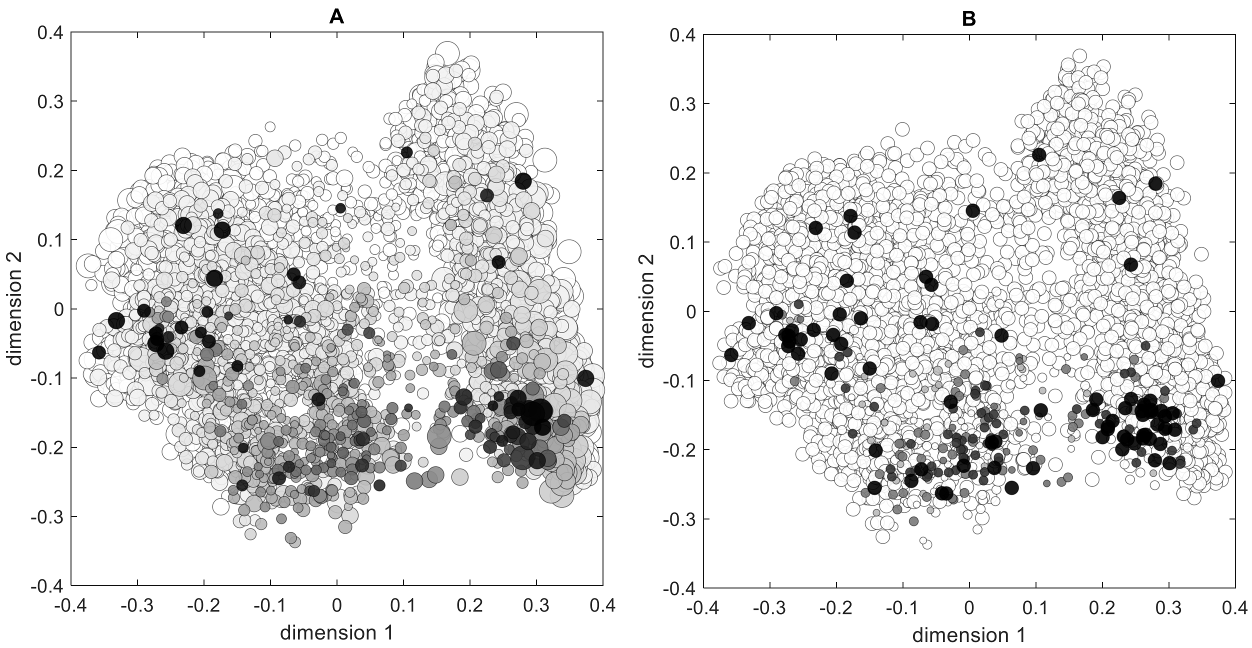

Figure S1. Plot of first and second multidimensional scaling dimensions for the agonism set; each point represents one chemical and it is colored on a grey scale representing the degree of misclassifications (A) of individual QSAR models and (B) of consensus strategies (the darker, the higher the number of misclassifications). The size of each point is proportional to the percentage of models (A) or consensus strategies (B) that provided a reliable prediction for the chemical.

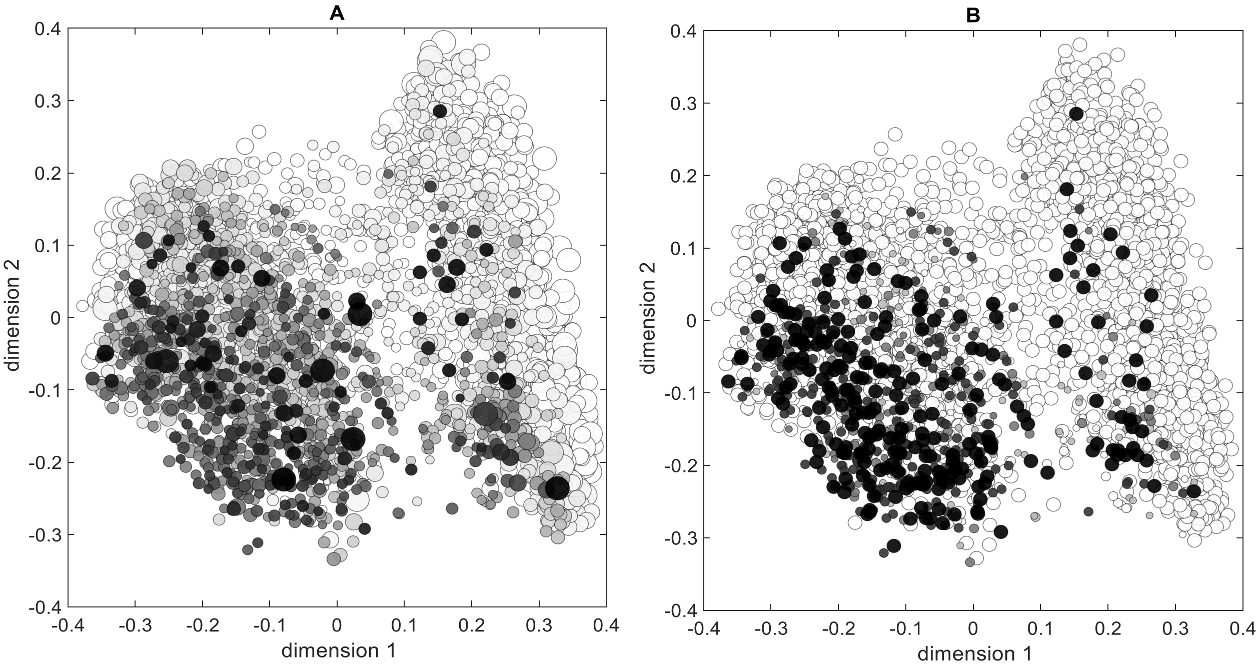

Figure S2. Plot of first and second multidimensional scaling dimensions for the antagonism set; each point represents one chemical and it is colored on a grey scale representing the degree of misclassifications (A) of individual QSAR models and (B) of consensus strategies (the darker, the higher the number of misclassifications). The size of each point is proportional to the percentage of models (A) or consensus strategies (B) that provided a reliable prediction for the chemical.

(1) Mansouri, K.; Kleinstreuer, N.; Abdelaziz, A.; Alberga, D.; Alves, V. M.; Andersson, Pa. L.; Andrade, C. H.; Bai, F.; Balabin, I.; Ballabio, D.; Wedebye, E. B.; Benfenati, E.; Bhhatarai, B.; Boyer, S.; Chen, J.; Consonni, V.; Farag, S.; Fourches, D.; Garcìa-Sosa, A. T.; Gramatica, P.; Grisoni, F.; Grulke, C. M.; Hong, H.; Horvath, D.; Hu, X.; Huang, R.; Jeliazkova, N.; Li, J.; Li, X.; Liu, H.; Manganelli, S.; Mangiatordi, G.; Maran, U.; Marcou, G.; Martin, T.; Muratov, E.; Nguyen, D.; Nicolotti, O.; Nikolov, G. N.; Norinder, U.; Papa, E.; Petitjean, M.; Piir, G.; Poroikov, V.; Qiao, X.; Richard, A. M.; Roncaglioni, A.; Ruiz, P.; Rupakheti, C.; Sakkiah, S.; Sangion, A.; Schramm, K; Selvaraj, C.; Shah, I.; Sild, S.; Sun, L.; Taboureau, O.; Tang, Y.; Tetko, I.; Todeschini, R.; Tong, W.; Trisciuzzi, D.; Tropsha, A.; VanDenDriessche, G.; Varnek, A.; Wang, Z.; Williams, A. J.; Xie, H.; Zakharov, A.; Zheng, Z.; Judson, R. S. CoMPARA: Collaborative Modeling Project for Androgen Receptor Activity. *Environ. Health Perspect.* **2020**, *128(2)*, 027002/1-17. https://doi.org/10.1289/EHP5580.
